# Supplementary material for: Cancer Metabolic Subtypes and Their Association with Molecular and Clinical Features
Source: Cancers (Basel). 2022 Apr 25;14(9):2145. doi: 10.3390/cancers14092145 (PMC9104370; doi:10.3390/cancers14092145)
Supplement: Supplementary file 1 [file cancers-14-02145-s001.zip › Supplementary Figures and Tables.pdf]

Supplementary Figures and Tables

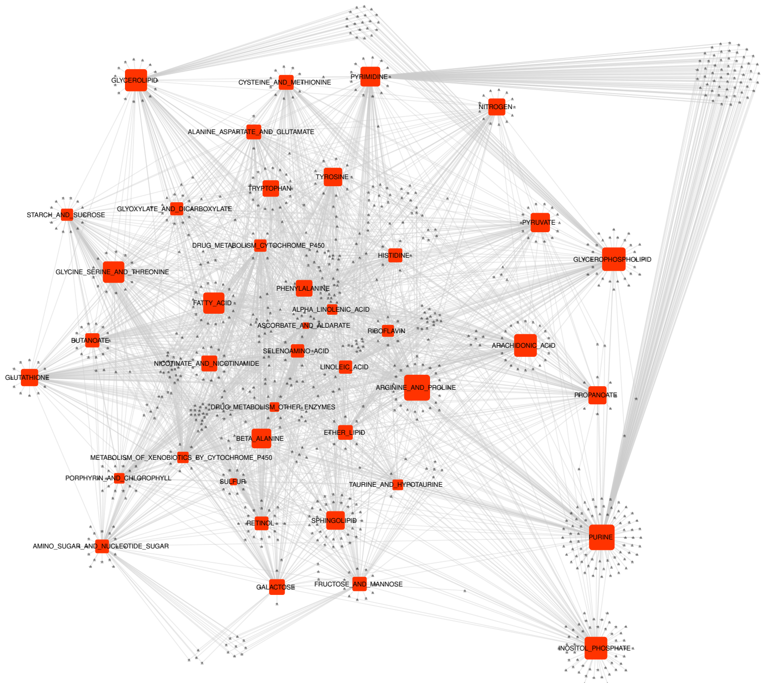

Figure S1: The KEGG pathways (red squares) used to cluster tumor samples and the respective genes (grey triangles) represented as a bipartite network. The size of the squares is proportional to the number of associated genes.

Table S1: Number of samples used for each tumor type.

ID	Tumor_type	Samples
ACC	Adrenocortical Carcinoma	79
BLCA	Bladder Urothelial Carcinoma	408
BRCA	Breast Invasive Carcinoma	1093
CESC	Cervical Squamous Cell Carcinoma and Endocervical Adenocarcinoma	304
CHOL	Cholangiocarcinoma	36
COAD	Colon Adenocarcinoma	285
COADREAD	(= COAD + READ)	379
DLBC	Lymphoid Neoplasm Diffuse Large B-cell Lymphoma	48
ESCA	Esophageal Carcinoma	184
GBM	Glioblastoma Multiforme	153
GBMLGG	(= GBM + LGG)	669
HNSC	Head and Neck Squamous Cell Carcinoma	520
KICH	Kidney Chromophobe	66
KIPAN	(= KICH + KIRC + KIRP)	889
KIRC	Kidney Renal Clear Cell Carcinoma	533
KIRP	Kidney Renal Papillary Cell Carcinoma	290
LAML	Acute Myeloid Leukemia	173
LGG	Brain Lower Grade Glioma	516
LIHC	Liver Hepatocellular Carcinoma	371
LUAD	Lung Adenocarcinoma	515
LUNG	(= LUAD + LUSC)	1016
LUSC	Lung Squamous Cell Carcinoma	501
MESO	Mesothelioma	87
OV	Ovarian Serous Cystadenocarcinoma	303
PAAD	Pancreatic Adenocarcinoma	178
PCPG	Pheochromocytoma and Paraganglioma	179
PRAD	Prostate Adenocarcinoma	497
READ	Rectum Adenocarcinoma	94
SARC	Sarcoma	259
SKCM	Skin Cutaneous Melanoma	103
STAD	Stomach Adenocarcinoma	415
STES	(= STAD + ESCA)	599
TGCT	Testicular Germ Cell Tumors	150
THCA	Thyroid Carcinoma	501
THYM	Thymoma	120
UCEC	Uterine Corpus Endometrial Carcinoma	176
UCS	Uterine Carcinosarcoma	57
UVM	Uveal Melanoma	80

Table S2: Molecular and phenotypic variables analyzed for association with metbolic subtypes

class	description	type
CNA	Copy number alterations	molecular
Methylation	Global DNA methylation	molecular
miRNA	microRNA expression	molecular
Mutations	Specific point mutations	molecular
Gene Mutations	Gene-level mutations	molecular
RPPA	Protein expression	molecular
Clinical	Clinical and histologic parameters	phenotypic
OS	Overall survival	phenotypic
RFS	Recurrence-free survival	phenotypic

Table S3: Top recurrent associations for each class of variables

Variable	Metabolism	Number of associations	Tumors associated
miRNA: hsa-mir-222	INOSITOL PHOSPHATE	12	BLCA, BRCA, CESC, HNSC, KIRC, LGG, LIHC, LUAD, SARC, STAD, TGCT, THCA
Methylation	GLYCEROLIPID	10	BLCA, CESC, COAD, HNSC, LGG, PCPG, SARC, TGCT, THCA, THYM
Methylation	INOSITOL PHOSPHATE	10	BLCA, COAD, KIRP, LGG, LIHC, LUAD, PCPG, TGCT, THCA, THYM
Methylation	PHENYLALANINE	10	BLCA, CESC, COAD, LGG, LIHC, LUAD, OV, PCPG, SARC, TGCT
Methylation	PURINE	10	BLCA, CESC, KIRP, LIHC, LUAD, PCPG, SARC, TGCT, THCA, THYM
Clinical: histological type	TYROSINE	8	BRCA, ESCA, LGG, PCPG, SARC, THCA, THYM, UCEC
RPPA: CCNB1|Cyclin B1	PURINE	7	BLCA, BRCA, KIRC, KIRP, LUAD, TGCT, THYM
CNA: chr17p12	FATTY ACID	6	COAD, KIRP, LGG, OV, PAAD, TGCT
CNA: chr17q11	GLYCEROLIPID	6	BRCA, LGG, LUSC, SARC, TGCT, THYM
CNA: chr17q21	GLYCEROLIPID	6	BRCA, HNSC, LGG, LUSC, TGCT, THYM
CNA: chr3p	INOSITOL PHOSPHATE	6	BLCA, BRCA, ESCA, KIRC, LUSC, UVM
CNA: chr3p14	INOSITOL PHOSPHATE	6	BLCA, BRCA, ESCA, KIRC, LUSC, UVM
CNA: chr3p21	ARGININE AND PROLINE	6	BRCA, ESCA, KIRC, PRAD, SARC, THYM
CNA: chr3p21	INOSITOL PHOSPHATE	6	BLCA, BRCA, ESCA, KIRC, LUSC, UVM
CNA: chr3q	PURINE	6	BRCA, ESCA, HNSC, LUSC, UCEC, UVM
CNA: chr3q24	PURINE	6	BRCA, ESCA, HNSC, LUSC, UCEC, UVM
CNA: chr3q25	PURINE	6	BRCA, ESCA, HNSC, LUSC, UCEC, UVM
CNA: chr3q26	PURINE	6	BRCA, ESCA, HNSC, LUSC, UCEC, UVM
CNA: chr3q27	PURINE	6	BRCA, ESCA, HNSC, LUSC, UCEC, UVM
CNA: chr3q28	FATTY ACID	6	BRCA, ESCA, HNSC, LGG, LUAD, LUSC
CNA: chr3q28	PURINE	6	BRCA, ESCA, HNSC, LUSC, UCEC, UVM
CNA: chr3q29	FATTY ACID	6	BRCA, ESCA, HNSC, LGG, LUAD, LUSC

Variable	Metabolism	Number of associations	Tumors associated
CNA: chr3q29	PURINE	6	BRCA, ESCA, HNSC, LUSC, UCEC, UVM
OS	PROPANOATE	2	KIRC, LGG
OS	PURINE	2	ACC, KIRC
OS	PYRIMIDINE	2	ACC, LGG
OS	SPHINGOLIPID	2	KIRC, LGG
